# Differences in Intestinal Flora and Its Related Metabolite Between Healthy and Diarrheal Piglets: Strategies for Innovative Bacterial Diarrhea Prevention

**DOI:** 10.3390/ani16101500

**Published:** 2026-05-14

**Authors:** Ling Zhang, Beiying Yang, Baosheng Liu, Shuang Xie, Ming Xing, Guoping Wu, Jinhua Zhang

**Affiliations:** 1 College of Animal Science and Technology, Jiangxi Agricultural University, Nanchang 330045, China; 18438615198@163.com (L.Z.);; 2 College of Food Science and Engineering, Jiangxi Agricultural University, Nanchang 330045, China

**Keywords:** piglet, bacterial diarrhea, intestinal flora, metabolites

## Abstract

The prevalence of piglet diarrhea poses a significant challenge to the development of pig farming, with disruptions in the intestinal flora recognized as a primary contributing factor. A comprehensive understanding of the intestinal flora composition in piglets is crucial for developing effective preventive strategies to reduce the incidence of diarrhea. In this study, we employed high-throughput sequencing and untargeted metabolomics via liquid chromatography–mass spectrometry (LC-MS) to investigate the differences in intestinal flora and their metabolites between healthy and diarrheal piglets. In this study, we found that *Lactobacillus* and *Enterococcus* were the predominant bacterial genera in healthy piglets. Amino acids, bioactive sesquiterpenoids, and triterpenoids were highly expressed in the intestines of healthy piglets. The association analysis between the microbiota and metabolites indicated that these metabolites showed a significant positive correlation with *Lactobacillus* and *Enterococcus*. Those results imply that *Lactobacillus* and *Enterococcus* might exert the functions of maintaining intestinal health and preventing diarrhea in piglets through these metabolites.

## Simple Summary

Piglet diarrhea is a major disease limiting the healthy farming of swine, often leading to severe economic losses and antibiotic overuse. In recent years, gut microbiota imbalance has been proven to be closely associated with the occurrence and development of various animal diseases. However, our understanding of the systematic differences between the gut microbiome and its metabolites in healthy versus diarrheic piglets remains limited. This study compared differences in the gut microbial community structure and metabolite composition between healthy and diarrheic piglets, focusing on the significant positive correlation between core functional microbial communities and beneficial metabolites (such as amino acids, sesquiterpenoids and triterpenoids) in the intestines of healthy piglets. This provides a robust data foundation for precisely screening next-generation microbial preparations with probiotic functions or metabolic intervention targets.

## 1. Introduction

Diarrhea in piglets poses a significant challenge to the pig industry, adversely impacting piglet growth and development, and in severe cases, leading to life-threatening outcomes. Various factors, including pathogenic infections, physiological imbalance, and environmental changes, can cause disturbance in the intestinal health of piglets, leading to diarrhea. The peri-weaning period is widely recognized as a critical stage associated with a high risk of diarrhea [1]. Previous studies have demonstrated that reduced gastric acid secretion in weaned piglets compromises protein digestion, leading to the accumulation of undigested nutrients in the gastrointestinal tract. These changes are associated with increased gastrointestinal pH and impaired digestive efficiency, which favor the proliferation of pathogenic microorganisms. Consequently, pathogens such as *Escherichia coli*, *Salmonella enterica*, and *Clostridium perfringens* can colonize the intestine and contribute to the development of diarrhea [2]. All in all, disturbances of the intestinal flora are considered to be a pivotal factor contributing to diarrhea in piglets [3].

The intestinal microbiota plays a pivotal role in supporting the host’s normal physiological functions. Microbial colonization of the intestinal tract facilitates the production of peptide-based antimicrobial agents, thereby enhancing the host’s defense mechanisms against pathogenic incursions [4]. The balance of intestinal flora of piglets is fragile and susceptible to various influencing factors. This microbial imbalance leads to the destabilization of the indigenous intestinal flora, culminating in the onset of diarrhea [3]. Furthermore, the administration of oral antibiotics, alterations in daily feed composition, and environmental condition changes can significantly disrupt the microbial homeostasis within the intestines, potentially leading to imbalances in the gut microbiota of piglets [5]. Antibiotics have been widely used to treat bacterial diarrhea in piglets. However, their overuse and misuse have caused issues such as bacterial resistance, environmental pollution, and drug residues. Additionally, improper antibiotic application disrupts the intestinal microbial balance in piglets, often leading to diarrhea [6]. These challenges highlight the urgent need for effective alternatives to antibiotics in managing intestinal diseases.

In addition to the composition of microorganisms, the metabolites produced by intestinal microorganisms are also key regulatory factors in the interaction between the host and microorganisms. These metabolites, including amino acids, short-chain fatty acids, and other bioactive compounds, play a crucial role in regulating immune responses, maintaining the integrity of epithelial cells, and regulating inflammation [7]. Therefore, a comprehensive analysis of the microbial community and metabolome can provide a deeper understanding of the functional consequences of microbial changes. However, it should be noted that omics-based methods can only identify candidate microbial species and related metabolites, but cannot directly determine probiotics. The classification of probiotics requires strict functional validation, safety assessment, and in vivo efficacy testing. In contrast, metabolites produced by microorganisms, such as bacteriocins and postbiotics, represent the functional output of microbial activities and may serve as promising targets for intervention strategies [8]. Postbiotic metabolites are compounds that regulate health and are produced by probiotics during the process of digestion and fermentation of dietary fibers [9]. Compared to active probiotics, postbiotic metabolites have advantages in safety, stability, and application convenience [10]. Therefore, studying metabolites related to the microbial community may provide new insights for the development of strategies for preventing and managing pig diarrhea based on postbiotic metabolites.

Although there is growing interest in the gut microbiota and metabolomics, there is still a lack of systematic studies on the analysis of microbiota and metabolomics in piglets under natural disease conditions (especially during the early stages of life). Therefore, this study aimed to investigate the differences in intestinal microbiota and fecal metabolite profiles between healthy and diarrheal piglets using 16S rRNA sequencing and LC-MS-based metabolomics. The main objective of this study is to identify the microbial and metabolic characteristics of healthy piglets, with a focus on understanding the microbial factors and metabolites that play a role in intestinal health. Although metabolic differences were observed between healthy and diarrheal piglets, the core goal is to study the microbial species and metabolites that promote health in healthy individuals. By integrating these data sets, we aim to identify potential microbial and metabolic features related to intestinal health, providing a basis for future functional studies and the development of interventions targeting the microbiota.

## 2. Materials and Methods

Fecal samples were collected from swine farms in Jiangxi Province, China. All the samples were collected from a single breeding farm under the same management, feeding, and environmental conditions. The evaluation criteria for the health or diarrhea of the piglets were based on the Hermann-Bank test criteria [11]. In total, 24 pre-weaning piglets (one-week-old) with the same rearing and immunization background (12 healthy piglets and 12 piglets with diarrhea) were selected from the same breeding center. The samples were collected from the rectum of the diseased or healthy piglets using cotton swabs. All of the collected samples were flash-frozen in liquid nitrogen immediately and subsequently stored at −80 °C. The sample information is shown in Table S1.

Total bacterial genomic DNA from the samples was extracted from the samples using the Tiangen Fecal DNA Extraction Kit (Tiangen, Beijing, China). The genomic DNA was used as a template for PCR amplification of the 16S rDNA V3-V4 region by selecting bacterial universal primers 338F and 907R. PCR reaction procedures and systems were described in the literature [12]. Nucleic acid electrophoresis was performed after PCR, and the results were observed. The DNA samples with clear and bright bands were sent to Majorbio (Shanghai Majorbio Bio-pharm Technology Co., Ltd., Shanghai, China) for sequencing analysis using the Miseq platform (Illumina, San Diego, CA, USA). Raw data were spliced according to overlap relationships, while the quality of the sequences was controlled and filtered, and the processed sequence information was used for subsequent analysis.

To further explore the relationship between the differential metabolites and their gut microbial community in healthy and diarrheic piglets, 12 samples (6 from the healthy group H and 6 from the diarrheal group D) were randomly selected from the aforementioned 24 samples for a metabolomics analysis. The LC-MS untargeted metabolomics method was employed to study the differences in intestinal microbial metabolites between the healthy and diarrheal piglets. In brief, 50 mg of fecal samples were transferred to 2 mL centrifuge tubes, a 6 mm grinding bead was added, and 400 μL of extraction solution (methanol/deionized water = 4:1, *v*/*v*) was added, including an internal standard extraction solution (l-2 chlorophenylalanine, 2 μg/mL). Then, the samples were ground for 6 min using a frozen tissue grinder (−10 °C, 50 Hz), and subsequently subjected to ultrasonic treatment in an ice-water bath (5 °C, 40 kHz). After centrifugation, the supernatant was collected in a glass bottle and dried using a vacuum concentrator at 37 °C. The dried samples were re-solubilized in 50% acetonitrile and then subjected to ultrasonic treatment and centrifugation again. A total of 75 μL of the supernatant was transferred to a new glass bottle for LC/MS analysis. The quality control (QC) samples were prepared by mixing equal amounts of the clear liquids from all the samples. The QC samples were processed and tested using the same methods as the analytical samples to represent the entire sample set, and were injected at fixed intervals to monitor the stability of the analytical process. The LC-MS/MS analysis of the samples was carried out on the Thermo UHPLC-Q Exactive HF-X system, equipped with an ACQUITY HSS T3 chromatographic column (100 × 2.1 mm inner diameter, 1.8 μm; Waters, Milford, MA, USA), completed by (Shanghai Meiji Biomedical Technology Co., Ltd., Shanghai, China). The mobile phase A was 0.1% formic acid aqueous solution:acetonitrile (95:5, *v*/*v*), and the mobile phase B was 0.1% formic acid acetonitrile:isopropanol:water (47.5:47.5:5, *v*/*v*). The flow rate was 0.40 mL/min, and the column temperature was 40 °C. The mass spectrometry (MS) analysis of the extract was conducted using the TripleTOF 6600 mass spectrometer (SCIEX, Framingham, MA, USA). The metabolites were identified by comparing with the original secondary mass spectrometry database provided by Shanghai Meiji Biomedical Technology Co., Ltd.

To clarify the physical and chemical properties and biological functions of metabolites, the online databases KEGG (http://www.kegg.jp/) and HMDB (http://www.hmdb.ca/) were used for primary and secondary identification and annotation of the metabolites.

The sequences were analyzed using the Uparse software (Uparse v7.0.1001, http://drive5.com/uparse/), and accessed on 26 September 2023. Sequences with a similarity of ≥97% were classified into the same operational taxonomic units (OTUs). Representative sequences of each OTU were selected, and species classification information was annotated based on the Mothur algorithm using the Silva database (https://www.arb-silva.de/).

The alpha diversity of the samples was analyzed using QIIME (Version 1.7.0), and the results were presented using the R software (Version 2.15.3). The alpha diversity analysis indicators included Sobs, Shannon, Simpson, Chao1, ACE, and Coverage. The result data were statistically analyzed using SPSS 22.0. Based on the clustering results of OTUs, the Majorbio Cloud Platform was used to analyze the number of common and unique OTUs between different groups, and the results were presented in the form of a Venn diagram. Based on the species annotation results, the species with higher abundance at the phylum and genus classification levels of each group were selected, and the cumulative bar chart of species relative abundance was drawn. Subsequently, similarity analysis (ANOSIM) was used to evaluate the statistical significance of the differences between groups and within groups.

The PLS-DA and OPLS-DA analyses were employed to visually represent the metabolic differences between group H and group D, and the models were evaluated through seven-fold cross-validation and response permutation tests.

Using the psych (v 2.3.12) package in R, the Pearson correlation coefficient between microbial taxonomic units and metabolites was calculated, and the statistical significance (*p* < 0.05) was determined through Fisher-Z transformation. Microbiota–metabolite pairs with an absolute correlation coefficient greater than 0.05 were selected for subsequent analysis. After the correlation analysis, redundant and unannotated metabolites were removed, and only the metabolites under the KEGG A category “metabolism” pathway and their corresponding microbial taxonomic units were retained.

Statistical analysis was conducted using R v4.3.2 (R Foundation for Statistical Computing, Vienna, Austria). All graphs and visualization results were generated using the ggplot2 (v 3.5.0) and ggcor (v 0.9.7) packages in R software. Descriptive statistical results were expressed as means ± standard deviations (SD), and statistical inferences were based on a significance level of α = 0.05.

## 3. Results

### 3.1. Microbial Community Characteristics of Healthy and Diarrheal Piglets

**Figure 1:**
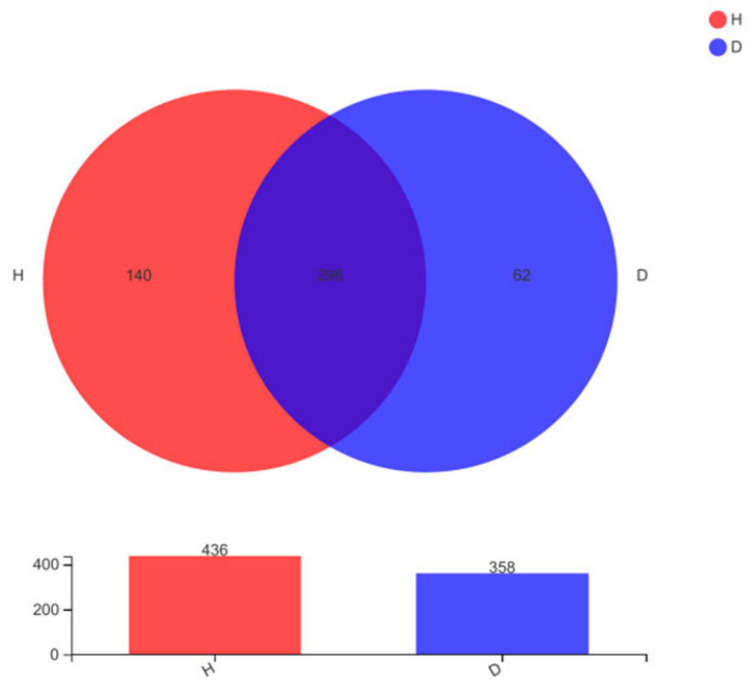
Venn diagram based on OTUs of the healthy piglets (H) and diarrheal piglets (D) groups.

A total of 1,080,505 high-quality sequences were obtained from all the samples after removing the barcodes and primers, as well as filtering out low-quality and chimeric reads. These high-quality sequences were clustered into 498 OTUs based on 97% similarity. In total, 296 OTUs were identified in the microbiota of the H and D groups, with 140 and 62 uniquely identified OTUs, respectively (Figure 1). The alpha diversity analysis revealed significant differences in the ACE and Chao1 index between the H and D groups, with *p*-values of 0.025 and 0.037, respectively. Specifically, the ACE index was 167.90 ± 46.66 in group H and 118.79 ± 53.18 in group D, while the Chao1 index was 169.46 ± 53.76 and 121.67 ± 51.49, respectively.

Based on the highly significant difference in the coverage index (*p* = 0.001), group H had a higher OTU richness than group D. The microbial diversity in group H was higher than that in group D when combined with the Sobs, Shannon and Simpson indices, but these differences were not statistically significant (*p* > 0.05) (Table 1).

### 3.2. Differences in Intestinal Flora Between Healthy and Diarrheal Piglets

**Figure 2:**
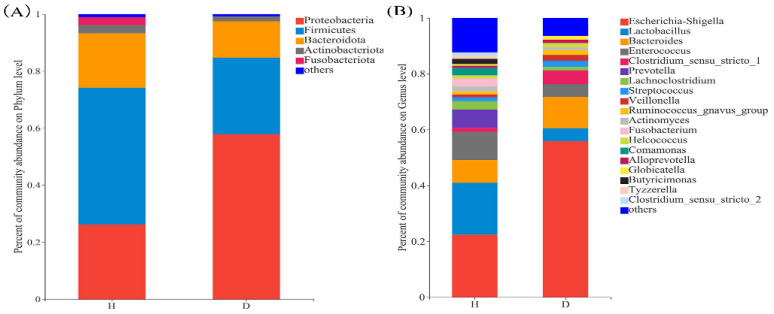
Comparison of the microbiota abundance among the healthy piglets (H) and diarrheal piglets (D): (**A**) The bar chart of the microbiota abundance among the two groups at the phylum level. (**B**) The bar chart of the microbiota abundance among the two groups at the genus level.

**Figure 3:**
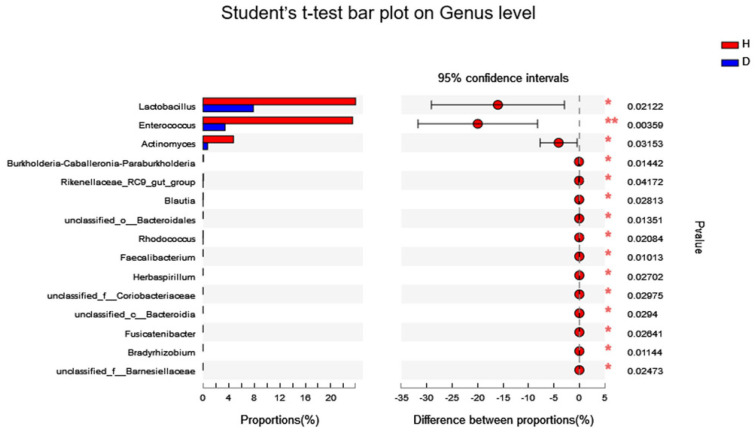
Phylotypes significantly different in gut microbiota of healthy piglets (H) and diarrheal piglets (D) at genus levels. Data of healthy and diarrheal groups were shown as relative abundance (%) of family and genus in each group. Statistical analysis was performed by Student’s *t*-test. *n* = 6, in each group. * *p* < 0.05, ** *p* < 0.001 healthy vs. diarrheal group.

At the phylum level, the intestinal flora of the healthy (H) and diarrheal (D) piglets exhibited similarities in the relative abundance of *Protebacteria*, *Firmicutes* and *Bacteroidota*. However, the relative abundance of *Firmicutes* was higher in group H (55.41%) compared to group D (26.85%), whereas *Bacteroidota* was significantly more abundant in group D (19.46%) than in group H (*p* < 0.05). Moreover, *Actinobacteriota* and *Fusobacteriota* were relatively more abundant in group H than in group D (Figure 2A). At the genus level, *Lactobacillus* (19.88% vs. 5.87%), *Enterococcus* (13.42% vs. 3.44%) and *Prevotella* (5.41% vs. 0.01%) showed higher relative abundances in group H compared to group D, whereas, *Escherichia-Shigella* (58.39% vs. 23.27%) and *Bacteroides* (13.30% vs. 0.09%) were more abundant in group D (Figure 2B). The genus-level analysis revealed significant differences between the two groups. Furthermore, the statistical analysis revealed significant differences in specific genera between the two groups. The relative abundances of *Enterococcus*, *Lactobacillus* and *Actinomyces* were significantly higher in group H than in group D (*p* < 0.05) (Figure 3).

### 3.3. Fecal Metabolome of Healthy and Diarrheal Piglets

**Figure 4:**
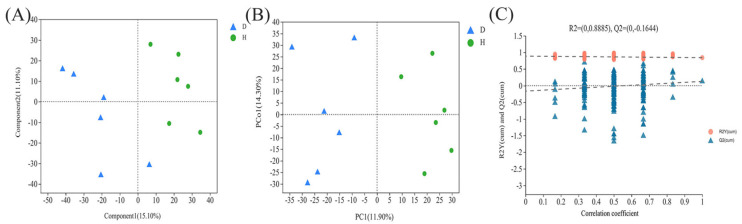
Metabolic profiles of the fecal samples from the H and D groups: (**A**) Partial least squares discriminant analysis. (**B**) Orthogonal partial least square discriminant analysis. (**C**) Permutation test of the OPLS-DA model for the healthy and diarrheal groups, The vertical axis represents the values of R2Y or Q2, and the two dotted lines respectively indicate the regression lines of R2Y and Q2.

The partial least squares discriminant analysis (PLS-DA) showed a distinct degree of separation between the samples from the H and D groups (Figure 4A). The orthogonal partial least square discriminant analysis (OPLS-DA) scores plot revealed significant differences between the two groups (Figure 4B), and the results of the displacement test, the values for R2Y (0.8885) and Q2Y (−0.1644 < 0) indicated the validity of the OPLS-DA model (Figure 4C).

**Figure 5:**
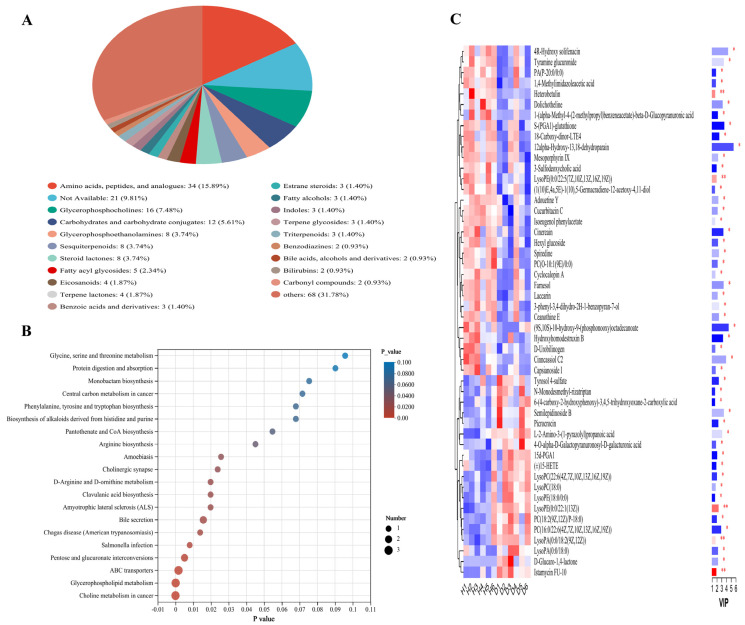
Difference in fecal metabolites between H and D groups: (**A**) Compound classification information on differential metabolites. (**B**) KEGG enrichment analysis. The abscissa indicates the enrichment significance *p*-value, and the ordinate indicates the KEGG pathway. The size of the bubbles represents the number of metabolites enriched in this pathway. (**C**) HCA. The abscissa indicates the name of the sample, and the ordinate indicates the differential metabolite. Different colors indicate metabolite expression abundance: the darker the color, the higher the expression abundance of the differential metabolite. *p*-value, * *p* < 0.05; ** *p* < 0.01.

In this study, a total of 1738 metabolites were identified, among which 544 metabolites were annotated in the Kyoto Encyclopedia of Genes and Genomes (KEGG) database. Through unpaired Student’s *t*-test (unpaired) and artificial matching searches, 182 differential metabolites were identified between the H and D groups (Table S2). The major components of these metabolites were amino acids, peptides, and analogues, glycerophosphocholines, carbohydrates and carbohydrate conjugates, glycerophosphoethanolamines and sesquiterpenoids (Figure 5A and Table S3). The identified differential metabolites were primarily associated with metabolic pathways such as pentose and glucuronate interconversions, ABC transporters, glycerophospholipid metabolism and choline metabolism in cancer (Figure 5B and Table S4). The hierarchical cluster analysis (HCA) of the top 50 significantly different metabolites revealed clear clustering patterns between group H and group D (Figure 5C). Notably, several metabolites, including heterobetulin, 1,4-Methylimidazoleacetic acid, 1-(alpha-Methyl-4-(2-methylpropyl) benzeneacetate)-beta-D-glucopyranuronic acid, S-(PGA1)-glutathione, 18-Carboxy-dinor-LTE4, lysoPE(0:0/22:5(7Z, 10Z, 13Z, 16Z, 19Z)), and 12alpha-Hydroxy-13,18-dehydroparain, showed higher relative abundance in group H compared to group D, indicating distinct metabolic features associated with intestinal health.

### 3.4. Characteristics of Metabolites in Healthy Piglets

**Figure 6:**
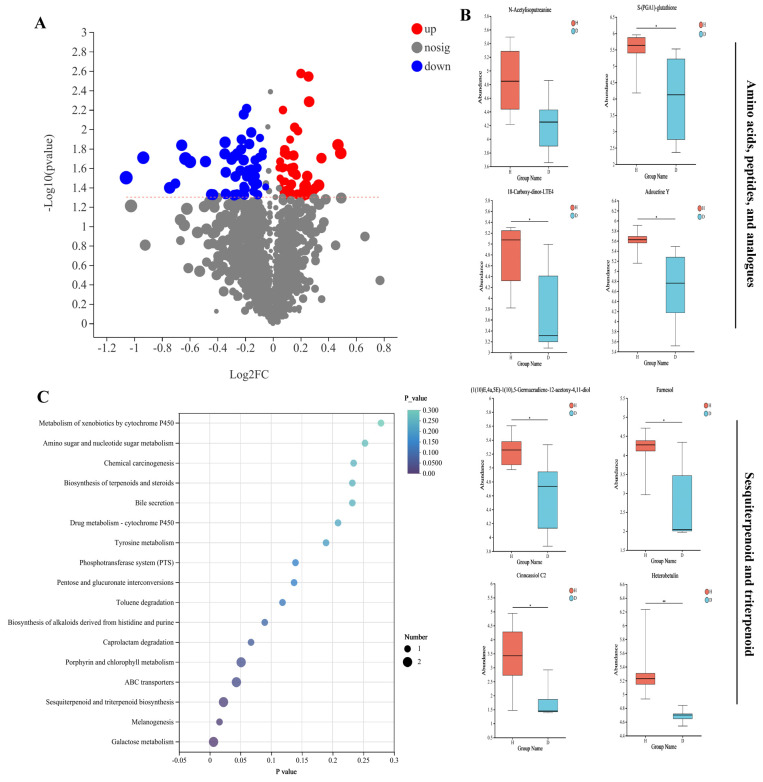
Characterization of highly expressed metabolites in group H: (**A**) Volcano plot of the H and D groups, where each point represents a metabolite. Red dots represent upregulated metabolites, blue dots represent downregulated metabolites, and gray dots indicate nonsignificant differences. (**B**) Eight differentially abundant metabolites involved in the main differential pathways were further illustrated by box maps. *p*-value, * *p* < 0.05; ** *p* < 0.01. (**C**) KEGG enrichment analysis. The abscissa indicates the enrichment significance *p*-value, and the ordinate indicates the KEGG pathway. The size of the bubbles represents the number of metabolites enriched in this pathway.

The differential metabolites analysis was conducted using the diarrheal group (D) as the reference. In comparison to group D, 51 metabolites were upregulated, while 34 metabolites were downregulated in group H (Figure 6A). The metabolites with higher expression levels in the healthy group consisted primarily of amino acids, peptides, and analogues (N-Acetylisoputreanine, S-(PGA1)-glutathione, 18-Carboxy-dinor-LTE4 and Adouetine Y), sesquiterpenoid and triterpenoid ((1(10)E,4a,5E)-1(10),5-Germacradiene-12-acetoxy-4,11-diol, farnesol, cinncassiol C2 and heterobetulin). The variations in the eight differentially enriched metabolites were further visualized using box maps (Figure 6B). In addition, 64 metabolites were detected exclusively in group H, while 51 metabolites were significantly upregulated in group H compared to group D (*p* < 0.05). These two groups partially overlapped, as some of the uniquely detected metabolites were also included among the significantly upregulated metabolites. Group D was characterized by a relative reduction in metabolites that were abundant in group H, particularly amino acid-related compounds, suggesting a state of metabolic imbalance associated with diarrhea.

To further explore the functional significance of these metabolites, the KEGG pathway enrichment analysis was performed on the upregulated metabolites in group H. The results showed that these metabolites were mainly involved in sesquiterpenoid and triterpenoid biosynthesis, melanogenesis, and galactose metabolism (Figure 6C). These pathways are closely related to host metabolism and immune regulation. For example, terpenoid-related pathways are associated with antimicrobial and anti-inflammatory activities, while galactose metabolism plays a role in energy supply and intestinal function.

### 3.5. Analysis of Association Between Gut Microbes and Their Metabolites in Healthy Piglets

**Figure 7:**
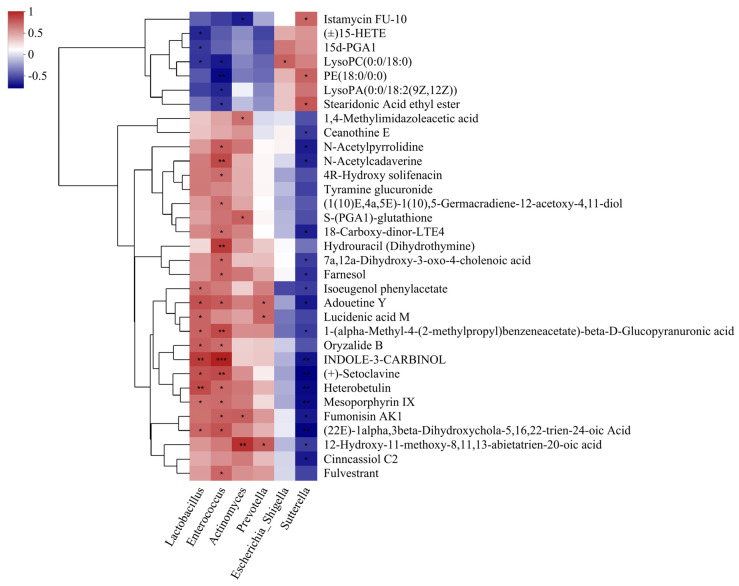
Correlations between metabolites and the differential genera of H and D groups. The right-hand side of the graph indicates the names of metabolites, and the bottom side indicates the names of differential genera of H and D groups. Each grid in the graph indicates the correlation between metabolites and differential genera: red represents a positive correlation and blue represents a negative correlation. *p*-value, * *p* < 0.05; ** *p* < 0.01; *** *p* < 0.001.

To further explore the microbiota–metabolite interactions in the healthy piglets, the predominant differential genera identified in the healthy and diarrheal piglets were selected, and Spearman’s correlation analysis was performed on the differential metabolites. As illustrated in Figure 7, amino acids (Adouetine Y), sesquiterpenoids (Farnesol) and indoles (INDOLE-3-CARBINOL) were positively correlated with *Enterococcus* and *Lactobacillus*, and negatively correlated with *Sutterella*. Moreover, 18-Carboxy-dinor-LTE4, (1(10)E,4a,5E)-1(10),5-Germacradiene-12-acetoxy-4,11-diol and bile acids (7a,12a-Dihydroxy-3-oxo-4-cholenoic acid) were also positively correlated with *Enterococcus*.

## 4. Discussion

Extensive research has demonstrated the critical role of the intestinal microbiota in maintaining host health and preventing pathogenic infections. Under normal physiological conditions, the intestinal tract harbors a diverse and dynamic community in a relatively stable equilibrium. Beneficial bacteria within the gut microbiota contribute to immune modulation, enhance defenses against pathogenic microorganisms, inhibit opportunistic pathogens, and support overall host health. Disruption of this microbial balance is widely recognized as a major factor contributing to diarrhea in piglets [3]. However, the specific impact of microbiota-derived metabolites on early-life piglet health and diarrhea remains insufficiently understood.

In the present study, we observed that the intestinal flora of the healthy piglets exhibited higher richness (ACE and Chao1, *p* < 0.05) compared to the diarrheal piglets, whereas no significant differences were found in diversity indices such as Sobs, Shannon and Simpson (*p* > 0.05). These results suggested that microbial richness, rather than overall diversity, may play a more important role in maintaining intestinal health in early-life piglets [11]. A significant difference in intestinal microbial composition was observed between the healthy and diarrheal piglets. In the healthy piglets, *Lactobacillus* and *Enterococcus* were notably more abundant, whereas *Escherichia-Shigella* and *Bacteroides* were more prevalent in the diarrheal piglets. These findings indicated that diarrhea may be associated with compositional imbalance rather than a simple loss of diversity.

Probiotics are defined as “live microorganisms when administered in adequate amounts confer a health benefit on the host” [13]. Lactic acid bacteria (LAB) are widely recognized as safe and are commonly used as probiotics to promote host health and improve intestinal function [14]. In this study, the higher abundance of LAB, particularly *Lactobacillus* and *Enterococcus*, in the healthy piglets is consistent with their known roles in suppressing pathogenic bacteria and improving gut function. Previous studies have shown that *Lacticaseibacillus rhamnosus* alleviates diarrhea induced by *Escherichia coli* K88 by modulating the intestinal flora and immune responses [15], while *Ligilactobacillus salivarius* enhances resistance to porcine epidemic diarrhea viruses (PEDV) [16]. Furthermore, *Lactiplantibacillus plantarum* has been demonstrated to be effective as a feed additive for reducing diarrhea in weaned piglets [17]. Similarly, *Enterococcus faecalis* EC-12 has been shown to effectively prevent diarrhea induced by *E. coli* [18]. These findings support our observation that enrichment of LAB is closely associated with intestinal health.

LAB contribute to host health not only through microbial interactions but also via the production of bioactive metabolites. In this study, 86 differential metabolites were identified between the two groups, among which 51 metabolites were significantly upregulated in the healthy piglets (*p* < 0.05). These metabolites mainly belonged to amino acids, peptides and their analogues, as well as sesquiterpenoids and triterpenoids. The enrichment of amino acid-related metabolites in the healthy piglets suggests an active metabolic environment supporting intestinal function, which may contribute to the enhanced intestinal resilience observed in the healthy group. Amino acids play a crucial role in promoting intestinal development and facilitating the repair of mucosal barrier damage by enhancing epithelial cell proliferation and differentiation [19]. Furthermore, functional amino acids contribute to the regulation of gene expression, translation, and signal transduction, thereby modulating inflammatory and oxidative responses as well as immune function [20]. The amino acid classes predominantly expressed in the intestines of the healthy piglets in this study were primarily Adouetine Y and 18-Carboxy-dinor-LTE4. Notably, Adouetine Y has demonstrated the capacity to inhibit the growth of Gram-positive and Gram-negative bacteria [21]. Sesquiterpenoids, characterized by their diverse structural frameworks and broad spectrum of biological activities, are regarded as promising candidates for the development of antibacterial and antifungal drugs. Over the past decades, numerous sesquiterpenoids with favorable antibacterial and antifungal properties have been successfully isolated from natural sources [22]. Furthermore, sesquiterpenoids and triterpenoids possess notable anti-inflammatory and antioxidant properties, which hold significant potential for pharmaceutical and industrial applications [23,24]. Farnesol, an acyclic sesquiterpene alcohol, has been identified as a highly abundant metabolite in the feces of healthy piglets. This compound has been reported to exhibit potent anti-cancer and anti-inflammatory activities [25]. The presence of such bioactive metabolites, with demonstrated antimicrobial and anti-inflammatory effects, contributes to the maintenance of a healthy intestinal microenvironment in piglets.

Importantly, correlation analysis revealed that several metabolites, including Adouetine Y, farnesol, heterobetulin, and indole-3-carbinol (I3C), were significantly positively correlated with *Lactobacillus* and *Enterococcus* (*p* < 0.01) and negatively correlated with *Sutterella*. These results suggest a potential functional link between beneficial bacteria and metabolite production. It is possible that *Lactobacillus* and *Enterococcus* either directly participate in or indirectly promote the production of these bioactive metabolites, thereby contributing to intestinal health. Notably, farnesol and heterobetulin are classified as triterpenoids and sesquiterpenoids, respectively. Research on the biological activity of heterobetulin is relatively limited, with the exception of its reported ability to increase urinary potassium excretion in rats [26]. In contrast, farnesol has been more extensively studied; for example, a study investigating its effects on CNS inflammatory demyelination in mice demonstrated that farnesol reshaped the dysbiotic gut microbiome, highlighting its potential role in modulating the gut–brain axis and contributing to its clinical activity [27]. Similarly, I3C is a bioactive phytochemical abundant in cruciferous vegetables, which possesses strong potential to prevent inflammation, cancer, and obesity [28]. In this study, we found that amino acids, peptides, and their analogues (e.g., Adouetine Y), as well as sesquiterpenoids and triterpenoids (e.g., farnesol and heterobetulin), were highly enriched in the healthy piglets and exhibited significant positive correlations with the abundance of *Lactobacillus* and *Enterococcus*. Given that sesquiterpenoids and triterpenoids are predominantly derived from plants and fungi, these findings suggest that *Lactobacillus* and *Enterococcus* in the intestinal tract may indirectly promote or directly participate in the synthesis of sesquiterpenoids and triterpenoids. This observation suggested that *Lactobacillus* and *Enterococcus* may play a role in the biosynthesis or metabolic regulation of sesquiterpenoids and triterpenoids within the intestinal environment.

In summary, our findings suggest that intestinal health in piglets is associated with a coordinated pattern of beneficial microbial enrichment and metabolite production, particularly involving amino acid-related metabolites and bioactive terpenoids. These results provide a foundation for future studies exploring microbiota-derived metabolites as potential targets for postbiotic-based strategies to improve gut health and prevent diarrhea. However, the composition of the intestinal microbiota is influenced by various factors (such as living environment, feeding conditions, etc.), so we need to further pay attention to the characteristics of the intestinal microbiota composition in different living environments of piglets.

## 5. Conclusions

This study investigated the differences in the intestinal flora and metabolites between healthy and diarrheal piglets. The findings revealed a higher abundance of *Lactobacillus* and *Enterococcus* in healthy piglets, accompanied by elevated levels of amino acids, peptides, and their analogues, as well as sesquiterpenoids and triterpenoids. These metabolites were significantly and positively correlated with the *Lactobacillus* and *Enterococcus*, suggesting that *Lactobacillus* and *Enterococcus* contribute to intestinal health not only through their antimicrobial effects but also by participating in the synthesis of amino acids, sesquiterpenoids, and triterpenoid metabolites. This study offers valuable insights into the role of intestinal microbiota and metabolites in maintaining gut health and provides a reference for strategies to prevent piglet diarrhea.

## Figures and Tables

**Table 1:** Differences in intestinal microbiota: Alpha diversity of the healthy and diarrheic piglets.

Item	H	D	*p* Value
Sobs	132.50 ± 36.82	99.83 ± 48.07	0.075
Shannon	2.21 ± 0.81	1.97 ± 0.71	0.442
Simpson	0.22 ± 0.17	031 ± 0.20	0.253
Chao1	169.46 ± 53.76	121.67 ± 51.49	0.037 *
ACE	167.90 ± 46.66	118.79 ± 53.18	0.025 *
Coverage	0.99 ± 0.00	0.99 ± 0.00	0.001 **

The data were expressed as the mean values ± standard deviation (SD). * *p* < 0.05, ** *p* < 0.001.

## Data Availability

The Illumina MiSeq sequences of bacterial 16S rRNA were submitted to GenBank-SRA under Bioproject PRJNA1013780 and PRJNA10013909. All untargeted metabolomic data used in this study have been deposited in the EMBL-EBI MetaboLights database with identifier MTBLS12406.

## Supplementary Materials

The following supporting information can be downloaded at https://www.mdpi.com/article/10.3390/ani16101500/s1. Table S1: Information of samples; Table S2: Annotated information on differential metabolites of feces between healthy piglets and piglets with diarrhea; Table S3: Compound classification information on differential metabolites; Table S4: KEGG Pathway information on differential metabolites.

## Abbreviations

The following abbreviations are used in this manuscript:

LC-MS	Liquid chromatography–mass spectrometry
QC	Quality control
OTUs	Operational taxonomic units
PLS-DA	Partial least squares discriminant analysis
OPLS-DA	Orthogonal partial least squares discriminant analysis
LAB	Lactic acid bacteria
PEDV	Porcine epidemic diarrhea viruses
