# Supplementary material for: Differences in Intestinal Flora and Its Related Metabolite Between Healthy and Diarrheal Piglets: Strategies for Innovative Bacterial Diarrhea Prevention
Source: Animals (Basel). 2026 May 14;16(10):1500. doi: 10.3390/ani16101500 (PMC13203167; doi:10.3390/ani16101500)
Supplement: Supplementary file 1 [file animals-16-01500-s001.zip › animals-4169673-supplementary.pdf]

Table. S1 Information of samples

Sample Type	Sample name	Group	Source
Feces	H1, H2, H3, H4, H5, H6, h3, h4, h5, h6, h7, h8	H	Piglet
	D1, D2, D3, D4, D5, D6, d1, d2, d4, d5, d6, d7	D	

Table. S2 Annotated information on differential metabolites of feces between healthy piglets and piglets with diarrhea

ID	Metabolite	KEGG Pathway Description	HMDB Superclass
pos_192	PC(16:0/0:0)[U]	-	-
pos_219	LysoPA(0:0/18:2(9Z,12Z))	-	Lipids and lipid-like molecules
pos_246	LysoPC(0:0/18:0)	-	Lipids and lipid-like molecules
pos_262	LysoPC(16:1(9Z))	Glycerophospholipid metabolism;Choline metabolism in cancer	-
pos_557	Lucidenic acid M	-	Phenylpropanoids and polyketides
pos_948	Stearidonic Acid ethyl ester	-	-
pos_1011	Bis(2-ethylhexyl) phthalate	-	-
pos_1037	PE(18:0/0:0)	-	-
pos_1072	1-Linoleoylglycerophosphocholine	-	Lipids and lipid-like molecules
pos_1324	Pyrimidine	-	Organoheterocyclic compounds
pos_1341	1,4-Methylimidazoleacetic acid	-	-
pos_1352	Crotanecine	-	-
pos_1498	Fumonisin AK1	-	Organic acids and derivatives
pos_2667	N-Acetylisoputreanine	-	Organic acids and derivatives
pos_3169	LysoPC(18:1(11Z))	Glycerophospholipid metabolism;Choline metabolism in cancer	Lipids and lipid-like molecules
pos_4581	(±)-Propionylcarnitine	-	-

pos_4797	PGP(16:1(9Z)/20:4(5Z,8Z,11Z,14Z))	-	Lipids and lipid-like molecules
pos_4798	Dolichotheline	Biosynthesis of alkaloids derived from histidine and purine;Biosynthesis of secondary metabolites	-
pos_4878	Tryptophyl-Histidine	-	Organic acids and derivatives
pos_4881	12-Hydroxy-11-methoxy-8,11,13-abietatrien-20-oic acid	-	Lipids and lipid-like molecules
pos_5050	Spiredine	-	-
pos_5101	Hypusine	-	Organic acids and derivatives
pos_5122	N-Monodesmethyl-rizatriptan	-	Organoheterocyclic compounds
pos_5229	Laccarin	-	Organoheterocyclic compounds
pos_5381	N1-(5-Phospho-a-D-ribosyl)-5,6-dimethylbenzimidazole	Metabolic pathways;Porphyrin and chlorophyll metabolism	Nucleosides, nucleotides, and analogues
pos_5494	Perindoprilat	-	Organic acids and derivatives
pos_5571	2H-Indol-2-one, 4-[2-(dipropylamino)ethyl]-1,3-dihydro-7-hydroxy-glucuronide	-	-
pos_5671	(3S,5R,6S,7E,9x)-7-Megastigmene-3,6,9-triol 9-glucoside	-	Lipids and lipid-like molecules
pos_5694	Sinapyl aldehyde	-	-
pos_5728	Fluocinolone Acetonide	-	Lipids and lipid-like molecules
pos_5797	PC(O-10:1(9E)/0:0)	-	-
pos_5848	Desogestrel	-	Lipids and lipid-like molecules

pos_5940	Coronafacic acid	-	-
pos_6011	NSC 23766	-	-
pos_6160	Bilirubin glucuronide	Metabolic pathways;Pentose and glucuronate interconversions;Bile secretion	Organoheterocyclic compounds
pos_6168	Tanakamine	-	Organoheterocyclic compounds
pos_6209	1-(alpha-Methyl-4-(2-methylpropyl)benzeneacetate)-beta-D-Glucopyranuronic acid	Metabolic pathways;Pentose and glucuronate interconversions;Bile secretion	Organic oxygen compounds
pos_6351	S-(PGA1)-glutathione	-	Organic acids and derivatives
pos_6386	7a,12a-Dihydroxy-3-oxo-4-cholenoic acid	-	Lipids and lipid-like molecules
pos_7374	Trilobacin	-	Lipids and lipid-like molecules
pos_7449	DG(15:0/16:1(9Z)/0:0)	-	Lipids and lipid-like molecules
pos_7606	Cucurbitacin C	-	Lipids and lipid-like molecules
pos_8260	Tetrahydroaldosterone-3-glucuronide	Metabolic pathways;Pentose and glucuronate interconversions;Bile secretion	Lipids and lipid-like molecules
pos_8363	D-Urobilinogen	Metabolic pathways;Biosynthesis of secondary metabolites;Porphyrin and chlorophyll metabolism	Organoheterocyclic compounds
pos_8388	Isoeugenol phenylacetate	-	Benzenoids
pos_8557	Methaqualone	-	Organoheterocyclic compounds
pos_8731	PC(16:0/22:6(4Z,7Z,10Z,13Z,16Z,19Z))	-	Lipids and lipid-like molecules
pos_10221	Phosphocholine	Metabolic pathways;Glycerophospholipid metabolism;Choline metabolism in cancer	-
pos_10257	PC(17:1(9Z)/0:0)	-	-
pos_10660	Desglucocoroloside	-	Lipids and lipid-like molecules
pos_11529	(22E)-1alpha,3beta-Dihydroxychola-5,16,22-trien-24-oic Acid	-	-

pos_11548	PA(P-20:0/0:0)	-	-
pos_11604	Coagulin R 3-glucoside	-	Lipids and lipid-like molecules
pos_11625	Oryzalide B	-	Lipids and lipid-like molecules
pos_11673	LysoPE(0:0/22:5(7Z,10Z,13Z,16Z,19Z))	-	Lipids and lipid-like molecules
pos_11709	Withaperuvin H	-	Lipids and lipid-like molecules
pos_11726	(1R,2R,4S)-p-Menthane-1,2,8-triol 8-glucoside	-	Organic oxygen compounds
pos_11779	Crocin 3	-	Lipids and lipid-like molecules
pos_11944	18-Carboxy-dinor-LTE4	-	Organic acids and derivatives
pos_11967	Cyclo(Leu-Phe)	-	Organic acids and derivatives
pos_12007	(1(10)E,4a,5E)-1(10),5-Germacradiene-12-acetoxy-4,11-diol	-	Lipids and lipid-like molecules
pos_12021	Benazeprilat	-	Organic acids and derivatives
pos_12073	Benzoylecgonine	-	-
pos_12074	Oxandrolone	-	Lipids and lipid-like molecules
pos_12118	4R-Hydroxy solifenacin	-	Organoheterocyclic compounds
pos_12143	3-phenyl-3,4-dihydro-2H-1-benzopyran-7-ol	-	Phenylpropanoids and polyketides
pos_12156	Cyclocalopin A	-	Organoheterocyclic compounds
pos_12168	Ichangic acid 17-beta-D-glucopyranoside	-	Lipids and lipid-like molecules
pos_12193	Sorbitan oleate	-	Lipids and lipid-like molecules
pos_12203	Cinereain	-	Organoheterocyclic compounds
pos_12221	10-Acetylpanaxytriol	-	Lipids and lipid-like molecules
pos_12233	Capsianoside I	-	Lipids and lipid-like molecules

pos_12264	INDOLE-3-CARBINOL	-	Organoheterocyclic compounds
pos_12279	Melibiose	ABC transporters;Galactose metabolism	Organic oxygen compounds
pos_12331	Tyrosyl-Proline	-	Organic acids and derivatives
pos_12344	N-Acetyl-leukotriene E4	-	Lipids and lipid-like molecules
pos_12444	Cinnamic acid	-	Phenylpropanoids and polyketides
pos_12501	N-Benzylformamide	-	-
pos_12611	Mifepristone	-	Lipids and lipid-like molecules
pos_12763	2-Methylbutyrylglycine	-	Organic acids and derivatives
pos_12823	[(5-oxo-1,7-diphenylheptan-2-yl)oxy]sulfonic acid	-	Phenylpropanoids and polyketides
pos_12946	12alpha-Hydroxy-13,18-dehydroparain	-	Lipids and lipid-like molecules
pos_12956	1,2,3,4,5,6-Hexahydro-5-(1-hydroxyethylidene)-7H-cyclopenta[b]pyridin-7-one	-	Organoheterocyclic compounds
pos_13197	MG(0:0/20:4(8Z,11Z,14Z,17Z)/0:0)	-	Lipids and lipid-like molecules
pos_13271	Ethoxyquin	Biosynthesis of secondary metabolites;Tropane, piperidine and pyridine alkaloid biosynthesis;Biosynthesis of alkaloids derived from ornithine, lysine and nicotinic acid	Organoheterocyclic compounds
pos_13493	Farnesol	Biosynthesis of terpenoids and steroids	Lipids and lipid-like molecules
pos_13549	Cinncassiol C2	-	Lipids and lipid-like molecules
pos_13591	Chitobiose	Metabolic pathways;Amino sugar and nucleotide sugar metabolism;Phosphotransferase system (PTS);ABC transporters	Organic oxygen compounds

pos_13860	3-(8,11,14-Pentadecatrienyl)phenol	-	Benzenoids
pos_14059	Cinncassiol C	-	Organic oxygen compounds
pos_14259	Dexpanthenol	Pantothenate and CoA biosynthesis	-
pos_14272	(S)C(S)S-S-Methylcysteine sulfoxide	-	Organic acids and derivatives
pos_14413	4-Chlorophenylacetic acid	Microbial metabolism in diverse environments;Tyrosine metabolism;DDT degradation	-
pos_14760	Hydrouracil (Dihydrothymine)	-	-
pos_14886	6-(4-carboxy-2-hydroxyphenoxy)-3,4,5-trihydroxyoxane-2-carboxylic acid	-	Organic oxygen compounds
pos_14930	N-Acetylcadaverine	-	Organic acids and derivatives
pos_15015	D-Glucaro-1,4-lactone	-	Organoheterocyclic compounds
pos_15063	Methylpyrazine	-	Organoheterocyclic compounds
pos_15161	N-Acetylpyrrolidine	-	-
pos_15351	Choline	Metabolic pathways;Glycerophospholipid metabolism;ABC transporters;Bile secretion;Glycine, serine and threonine metabolism;Cholinergic synapse;Choline metabolism in cancer	Organic nitrogen compounds
pos_15540	Istamycin FU-10	Biosynthesis of antibiotics	-
neg_178	Threoninyl-Glutamate	-	Organic acids and derivatives
neg_204	3-Dehydroquinate	Metabolic pathways;Biosynthesis of antibiotics;Phenylalanine, tyrosine and tryptophan biosynthesis;Biosynthesis of secondary metabolites;Biosynthesis of amino acids	Organic acids and derivatives
neg_795	(9S,10S)-10-hydroxy-9-(phosphonooxy)octadecanoate	-	Lipids and lipid-like molecules

neg_1108	(+)-Setoclavine	-	Alkaloids and derivatives
neg_1135	Corchorusoside C	-	Lipids and lipid-like molecules
neg_1333	Fructosamine	-	Organic oxygen compounds
neg_1345	PE(P-16:0e/0:0)	-	Lipids and lipid-like molecules
neg_2314	4-O-alpha-D-Galactopyranuronosyl-D-galacturonic acid	Metabolic pathways;Pentose and glucuronate interconversions;ABC transporters	Organic oxygen compounds
neg_2650	Semilepidinoside B	-	Organic oxygen compounds
neg_2682	2-Amino-5,6-dichloro-3,4,-dihydroquinazoline	-	Organoheterocyclic compounds
neg_2771	N-(5-Amino-2-hydroxybenzoyl)glycine	-	Benzenoids
neg_2976	5-ethyl-5-methyl-2,4-oxazolidinedione	-	Organoheterocyclic compounds
neg_3240	Capsidiol	Sesquiterpenoid and triterpenoid biosynthesis	Lipids and lipid-like molecules
neg_3584	4-[(Hydroxymethyl)nitrosoamino]-1-(3-pyridinyl)-1-butanone	Chemical carcinogenesis;Metabolism of xenobiotics by cytochrome P450	Organic oxygen compounds
neg_3830	2-(Arabinosylamino)-3-(glucosylamino)propanenitrile	-	Organic oxygen compounds
neg_3874	(4-{[2-methoxy-4-(prop-2-en-1-yl)phenoxy]carbonyl}phenyl)oxidanesulfonic acid	-	Phenylpropanoids and polyketides

neg_4028	Kanzonol R	-	Phenylpropanoids and polyketides
neg_4039	N-Acetyl desmethyl frovatriptan	-	Organoheterocyclic compounds
neg_4291	(±)-(E)-13-Hydroxy-10-oxo-11-octadecenoic acid	-	Lipids and lipid-like molecules
neg_4565	3-O-Acetylepisamarcandin	-	Phenylpropanoids and polyketides
neg_4596	Sarcodon scabrosus Depsipeptide	-	Organic acids and derivatives
neg_4622	(Z)-2,4-Dihydroxy-6-(8-pentadecenyl)benzoic acid	-	Benzenoids
neg_5202	LysoPC(22:6(4Z,7Z,10Z,13Z,16Z,19Z))	Glycerophospholipid metabolism;Choline metabolism in cancer	Lipids and lipid-like molecules
neg_5272	LysoPE(20:4(5Z,8Z,11Z,14Z)/0:0)	-	Lipids and lipid-like molecules
neg_5423	N-Palmitoyl tyrosine	-	Organic acids and derivatives
neg_5503	LysoPC(18:0)	Glycerophospholipid metabolism;Choline metabolism in cancer	Lipids and lipid-like molecules
neg_5511	8,11-eicosadiynoic acid	-	-
neg_5580	Heterobetulin	-	Lipids and lipid-like molecules
neg_5910	3-(Acetyloxy)-2-hydroxypropyl octadecanoate	-	Lipids and lipid-like molecules
neg_6028	Hexyl glucoside	-	Lipids and lipid-like molecules
neg_6062	LysoPE(0:0/22:2(13Z,16Z))	-	Lipids and lipid-like molecules

neg_6168	PC(18:2(9Z,12Z)/P-18:0)	-	Lipids and lipid-like molecules
neg_6197	Ceanothine E	-	Organic acids and derivatives
neg_6953	Adouetine Y	-	Organic acids and derivatives
neg_7018	Fulvestrant	-	Lipids and lipid-like molecules
neg_7089	PE(20:4(5Z,8Z,11Z,14Z)/P-18:1(11Z))	-	Lipids and lipid-like molecules
neg_7103	Mesoporphyrin IX	-	Organoheterocyclic compounds
neg_7115	LysoPA(0:0/18:0)	-	Lipids and lipid-like molecules
neg_7159	Ganoderic acid K	-	Lipids and lipid-like molecules
neg_7183	PC(18:1(11Z)/18:2(9Z,12Z))	-	Lipids and lipid-like molecules
neg_7222	PC(16:0/20:4(5Z,8Z,11Z,14Z))	Metabolic pathways;Glycerophospholipid metabolism;Biosynthesis of secondary metabolites;Arachidonic acid metabolism;Linoleic acid metabolism;alpha-Linolenic acid metabolism;Choline metabolism in cancer;Retrograde endocannabinoid signaling	Lipids and lipid-like molecules
neg_7231	PC(18:1(11Z)/20:4(5Z,8Z,11Z,14Z))	-	Lipids and lipid-like molecules
neg_7386	1-Stearoylglycerophosphoserine	-	Lipids and lipid-like molecules
neg_7642	LysoPE(0:0/22:1(13Z))	-	Lipids and lipid-like molecules
neg_7757	Hemorphin-4	-	Organic acids and derivatives
neg_7883	LysoPE(18:0/0:0)	-	Lipids and lipid-like molecules
neg_7950	Mulberrin	-	Phenylpropanoids and polyketides
neg_8009	Hydroxybuprenorphine	-	Benzenoids
neg_8136	LysoPC(15:0)	Glycerophospholipid metabolism;Choline metabolism in cancer	Lipids and lipid-like molecules
neg_8192	Bilastine	-	Organoheterocyclic compounds

neg_8254	LysoPC(18:2(9Z,12Z))	-	Lipids and lipid-like molecules
neg_8270	(±)15-HETE	-	-
neg_8392	15d-PGA1	-	-
neg_8845	(3b,20R,22R)-3,20,27-Trihydroxy-1-oxowitha-5,24-dienolide 3-glucoside	-	Lipids and lipid-like molecules
neg_8910	Hydroxyhomodestruxin B	-	Organic acids and derivatives
neg_9014	11-nitro-1-undecene	-	Organic 1,3-dipolar compounds
neg_9179	S-(11-OH-9-deoxy-delta9,12-PGD2)-glutathione	-	Organic acids and derivatives
neg_9224	Palmitoyl glucuronide	Metabolic pathways;Pentose and glucuronate interconversions;Bile secretion	Lipids and lipid-like molecules
neg_9230	P-Acetaminobenzoic acid	-	-
neg_9262	Corchorusoside A	-	Lipids and lipid-like molecules
neg_9298	Allodesmosine	-	Organic acids and derivatives
neg_9566	3-Sulfodeoxycholic acid	-	Lipids and lipid-like molecules
neg_9595	3-Benzoyloxy-6-oxo-12-ursen-28-oic acid	-	Lipids and lipid-like molecules
neg_9662	Nepetalactam	-	Organoheterocyclic compounds
neg_9883	4-Hydroxy-4-(3-pyridyl)-butanoic acid	Chemical carcinogenesis;Metabolism of xenobiotics by cytochrome P450	Organoheterocyclic compounds
neg_9901	Glutamylleucine	-	Organic acids and derivatives
neg_9902	Ethyl vanillin isobutyrate	-	Benzenoids
neg_9961	Epsilon-caprolactam	Metabolic pathways;Caprolactam degradation;Microbial metabolism in diverse environments	Organoheterocyclic compounds
neg_10027	9-Pentadecenoic acid	-	Lipids and lipid-like molecules
neg_10223	Bakkenolide C	-	Lipids and lipid-like molecules

neg_10284	Sinapoylputrescine	-	Phenylpropanoids and polyketides
neg_10384	Isoacitretin	-	Lipids and lipid-like molecules
neg_10680	Longistylin A	-	Phenylpropanoids and polyketides
neg_10730	Nandrolone	-	Lipids and lipid-like molecules
neg_10796	4-methoxybenzene-1,3-diol	-	Benzenoids
neg_10851	Tyramine glucuronide	Metabolic pathways;Pentose and glucuronate interconversions;Bile secretion	Organic oxygen compounds
neg_10903	Alitame	-	Organic acids and derivatives
neg_11457	Picrocrocin	-	Organic oxygen compounds
neg_12545	Tyrosol 4-sulfate	-	Organic acids and derivatives
neg_12652	L-Arginine	Metabolic pathways;Chagas disease (American trypanosomiasis);Arginine biosynthesis;Biosynthesis of secondary metabolites;Amyotrophic lateral sclerosis (ALS);Amoebiasis;D-Arginine and D-ornithine metabolism;ABC transporters;Arginine and proline metabolism;Clavulanic acid biosynthesis;Biosynthesis of amino acids;Protein digestion and absorption;Aminoacyl-tRNA biosynthesis;Monobactam biosynthesis;Biosynthesis of antibiotics;Central carbon metabolism in cancer;Biosynthesis of alkaloids derived from ornithine, lysine and nicotinic acid;Salmonella infection;Biosynthesis of plant secondary metabolites	Organic acids and derivatives
neg_12732	L-2-Amino-3-(1-pyrazolyl)propanoic acid	-	Organic acids and derivatives

Table. S3 Compound classification information on differential metabolites

Class	Number
Carboxylic acids and derivatives	38
Glycerophospholipids	29
Prenol lipids	22
Steroids and steroid derivatives	17
Fatty Acyls	16
Organooxygen compounds	14
Indoles and derivatives	5
Benzene and substituted derivatives	4
Pyridines and derivatives	4
Tetrapyrroles and derivatives	3
Peptidomimetics	3
Diazines	3
Coumarins and derivatives	3
Phenols	3
Flavonoids	3
Phenol esters	2
Glycerolipids	2
Organonitrogen compounds	2
Isoflavonoids	2
Purine nucleosides	2
Pyrrolopyridines	2
Quinolines and derivatives	2
Hydroxy acids and derivatives	2
Furopyrans	2
Diazanaphthalenes	2
Cinnamic acids and derivatives	2
Depsides and depsidones	1
Tetrahydroisoquinolines	1
Stilbenes	1
Azoles	1
Sphingolipids	1
Saccharolipids	1
Azolidines	1
Benzimidazole ribonucleosides and ribonucleotides	1
Benzimidazoles	1
Benzopyrans	1
Carboximidic acids and derivatives	1
Phenanthrenes and derivatives	1
Ergoline and derivatives	1
Diarylheptanoids	1
Diazinanes	1

Organic sulfuric acids and derivatives	1
Macrolides and analogues	1
Lactones	1
Lactams	1
Keto acids and derivatives	1
Dihydrofurans	1
Endocannabinoids	1
Indanes	1
Anthracenes	1
Allyl-type 1,3-dipolar organic compounds	1

Table. S4 KEGG Pathway information on differential metabolites

First Category	Second Category	Metabolite
Environmental Information Processing	Membrane transport	Digalacturonate; Melibiose; Chitobiose; Choline; 4-O-alpha-D-Galactopyranuronosyl-D-galacturonic acid; L-Arginine
Genetic Information Processing	Translation	L-Arginine
Human Diseases	Cancers: Overview	LysoPC(16:1(9Z)); LysoPC(18:1(11Z)); Phosphocholine; Choline; 4-[(Hydroxymethyl)nitrosoamino]-1-(3-pyridinyl)-1-butanone; LysoPC(22:6(4Z,7Z,10Z,13Z,16Z,19Z)); LysoPC(18:0); LysoPC(15:0); PC(16:0/20:4(5Z,8Z,11Z,14Z)); L-Arginine; 4-Hydroxy-4-(3-pyridyl)-butanoic acid
Human Diseases	Infectious diseases: Bacterial	L-Arginine
Human Diseases	Infectious diseases: Parasitic	Phosphatidylserine; L-Arginine
Human Diseases	Neurodegenerative diseases	L-Arginine
Metabolism	Amino acid metabolism	4-Chlorophenylacetic acid; Choline; Phosphatidylserine; L-Arginine; 3-Dehydroquinate;
Metabolism	Biosynthesis of other secondary metabolites	Ethoxyquin; L-Arginine
Metabolism	Carbohydrate metabolism	1-(alpha-Methyl-4-(2-methylpropyl)benzeneacetate)-beta-D-Glucopyranuronic acid; Tetrahydroaldosterone-3-glucuronide; Digalacturonate; Palmitoyl glucuronide; Tyramine glucuronide; Bilirubin glucuronide; Chitobiose; Melibiose; 4-O-alpha-D-Galactopyranuronosyl-D-galacturonic acid
Metabolism	Chemical structure transformation maps	Dolichotheline; Ethoxyquin; Farnesol; L-Arginine;

Metabolism	Lipid metabolism	LysoPC(16:1(9Z)); LysoPC(18:1(11Z)); Glycocholic acid; Choline; Phosphocholine; LysoPC(22:6(4Z,7Z,10Z,13Z,16Z,19Z)); LysoPC(18:0); PC(16:0/20:4(5Z,8Z,11Z,14Z)); LysoPC(15:0)
Metabolism	Metabolism of cofactors and vitamins	N1-(5-Phospho-a-D-ribosyl)-5,6-dimethylbenzimidazole; Dexpanthenol; D-Urobilinogen;
Metabolism	Metabolism of other amino acids	L-Arginine
Organismal Systems	Digestive system	1-(alpha-Methyl-4-(2-methylpropyl)benzeneacetate)-beta-D-Glucopyranuronic acid; Bilirubin glucuronide; Glycocholic acid; Choline; Tetrahydroaldosterone-3-glucuronide; L-Arginine; Tyramine glucuronide; Palmitoyl glucuronide
Organismal Systems	Nervous system	Choline; PC(16:0/20:4(5Z,8Z,11Z,14Z))
